# A Method for Quantifying Consistency in Animal Distributions Using Survey Data

**DOI:** 10.1371/journal.pone.0044353

**Published:** 2012-09-05

**Authors:** Joel P. Heath, William A. Montevecchi, Daniel Esler

**Affiliations:** 1 Mathematical Biology, Department of Mathematics, University of British Columbia, Vancouver, British Columbia, Canada; 2 Cognitive and Behavioural Ecology Programme, Memorial University of Newfoundland, St. John’s, Newfoundland, Canada; 3 Centre for Wildlife Ecology, Simon Fraser University, Delta, British Columbia, Canada; California State University Fullerton, United States of America

## Abstract

The degree of consistency with which groups of animals use the landscape is determined by a variety of ecological processes that influence their movements and patterns of habitat use. We developed a technique termed *Distributional Consistency* that uses survey data of unmarked individuals to quantify temporal consistency in their spatial distribution, while accounting for changes in population size. Distributional consistency is quantified by comparing the observed distribution patterns to all theoretically possible distribution patterns of observed individuals, leading to a proportional score between 0 and 1, reflecting increasingly consistent use of sites within a region. The technique can be applied to survey data for any taxa across a range of spatial and temporal scales. We suggest ways in which distributional consistency could provide inferences about the dispersal and habitat decisions of individuals, and the scales at which these decisions operate. Distributional consistency integrates spatial and temporal processes to quantify an important characteristic of different habitats and their use by populations, which in turn will be particularly useful in complimenting and interpreting other ecological measures such as population density and stability. The technique can be applied to many existing data sets to investigate and evaluate a range of important ecological questions using simple survey data.

## Introduction

Understanding patterns of distribution and abundance of organisms are basic concerns in ecology and wildlife conservation [1]. Time series of abundance and spatial distribution patterns have been primary areas of investigation. Interpretation of these data has important implications for understanding population dynamics and ecological niches, and in more applied contexts, population viability and habitat requirements.

Spatial and temporal patterns of distribution and abundance are often investigated independently, though these population features are closely linked. Distributional patterns change over time and are influenced by both changes in overall abundance and the movement of individuals among habitats. Here we present a method we term Distributional Consistency that incorporates both spatial and temporal components, allowing researchers to quantify how consistently a population within a given region is distributed among an array of sites, while controlling for changes in regional abundance. This provides a useful means to quantify spatiotemporal occupancy patterns that can be calculated at any desired spatial or temporal scale using simple survey data. In particular we explore its utility for evaluating differences in habitat use and relationships between individual decisions and larger scale distributional patterns.

A variety of techniques such as mark-recapture have evaluated the consistency with which individuals use particular sites in the context of philopatry and site fidelity (see [2]). Yet, it remains an important challenge to understand how behavioural decisions about habitat selection and movement influence processes at the landscape scale [3], [4]. Philopatry and dispersal decisions can be affected by proximate factors including an individual’s state or condition, the habitat attributes of potential sites, densities of conspecifics, competitors, and/or predators and can be influenced by past experience [5]–[8]. Philopatry/fidelity (hereafter fidelity) decisions of individuals will cumulatively influence distribution patterns observed at a regional level (see [3]). Stability of these patterns can therefore be used to make inferences about average movement decisions of individuals in the population. Understanding and measuring distributional consistency is therefore an important step in linking individual movements to population and landscape processes.

We introduce a measure of distributional consistency that can be calculated from simple, spatially explicit survey data of unmarked individuals, and can be applied across a range of spatial and temporal scales. We present the methods for calculating distributional consistency, then 1) discuss defining ‘sites’ and ‘regions’ over a range of scales, 2) assess the robustness of distributional consistency to survey error, and 3) propose that measuring the stability of spatiotemporal distribution patterns can provide insight into the ecological processes underlying demographic structure, including decisions individuals make about habitat use over time, and therefore provides an important new tool for informing conservation and wildlife managers.

## Methods

### Quantifying Distributional Consistency, DC

We define distributional consistency (DC) as the degree of temporal stability with which a group of individuals occupy an array of sites within a region. Although the consistency of a distribution among sites will on average reflect how individual sites are used, this does not imply that a regional measure of distributional consistency can be obtained by simply averaging occupancy rates across sites. In particular, mean occupancy rates often will decrease with decreasing population size due to inclusion of lower quality territories or habitats that are occupied only in high density years (and therefore have a low inter-annual occupancy rate; [9], [10]). Individuals that disperse from a region or die obviously can not consistently use a site; however, consistency in the population distribution could be maintained if recruits or immigrants preferentially settle at vacated sites. To facilitate comparisons among populations of different average abundances, and to account for how inter-annual changes in population size influence observed occupancy patterns, population size must be explicitly considered and accounted for at each time interval when evaluating consistency in the use of an array of sites.

Our approach to quantifying the consistency of a distribution pattern first involves determining how consistent it *could* be. This provides a standard against which to compare observed patterns. We use combinations/permutations statistics [11] to compute possible distribution patterns, given the observed changes in regional population size. Particularly because population size can change across time steps, the mathematical notation needed to describe the calculation may appear complicated at first. The concept is relatively simple however: how consistent is the distribution of observed individuals compared to other ways in which they could have been distributed. To conceptually illustrate how the technique works, we have included a series of tables that illustrate a sample calculation using simulated survey data.

To calculate distributional consistency, raw survey data are organized as a matrix of the number of individuals at each site (columns) in each time step (rows). Regional population size at each time step is therefore the sum for each row. While a strength of the technique is its ability to explicitly consider the number of individuals at each site, we start with a simple example, considering hypothetical data for a territorial species, when the individuals/pairs at each site in each time step can be 1 or 0, for presence/absence (Table 1). Any time step relevant to the species’ natural history could be used; we use one year as a generic time step for ease of discussion. In this example (territorial species; maximum of one individual or pair per site), the sum of each column is the number of years a site is occupied. If divided by years surveyed, this would be the site occupancy rate of Sergio and Newton [10] or could be interpreted as a presence/absence score as used in occupancy modelling [12]. In contrast, our approach can consider total abundance as well as presence/absence data, and goes beyond occupancy modelling by considering consistency among sites within a region, while accounting for changes in population size. Below the sum, at the bottom of Table 1, we have added a summary that indicates the level of consistency with which each site (column) was used (here ***k*** = 2, 3 or 4 years of consistency; note there is no ***k*** = 1 as a site used in only one year is not considered consistent use). Therefore, the sum of each of these rows is the total observed instances of ***k***-level consistency for the region, which we define as *O_k_*. When dealing with non-territorial species or different spatial scales, any number of individuals could occupy a given site during each time step. This does not influence the calculation of distributional consistency, but we defer further discussion of scenarios with multiple animals per site to a later section on defining the site and region.

**Table 1:** Survey matrix of the number of individuals at each site in each time step for a territorial species, used in the example calculation in the text.

Time	Site																Popn
	1	2	3	4	5	6	7	8	9	10	11	12	13	14	15	16	Size
**1**	1	0	0	0	1	1	1	1	0	0	0	1	1	1	0	1	**9**
**2**	0	1	0	0	1	1	0	0	1	1	0	1	1	0	1	1	**9**
**3**	0	0	1	0	0	0	1	0	1	0	1	1	0	1	1	1	**8**
**4**	0	0	0	1	0	0	0	1	0	1	1	0	1	1	1	1	**8**
**SUM**	1	1	1	1	2	2	2	2	2	2	2	3	3	3	3	4	
**Summary of Observed Consistency**																	**O_k_**
***k*** ** = 2**	*0*	*0*	*0*	*0*	*1*	*1*	*1*	*1*	*1*	*1*	*1*	*0*	*0*	*0*	*0*	*0*	***7***
***k*** ** = 3**	*0*	*0*	*0*	*0*	*0*	*0*	*0*	*0*	*0*	*0*	*0*	*1*	*1*	*1*	*1*	*0*	***4***
***k*** ** = 4**	*0*	*0*	*0*	*0*	*0*	*0*	*0*	*0*	*0*	*0*	*0*	*0*	*0*	*0*	*0*	*1*	***1***

Population size in each year is the sum of each row. Below the main table, the column sums and a summary of the number of instances a site was used to a given level of consistency (***k***) are presented, as described in the text.

For clarity, the extremes of ‘completely consistent’ and ‘completely inconsistent’ distributions are illustrated in Table 2, for two hypothetical populations, with 4 individuals in each of 4 years, over an array of 16 sites (note there are many combinations of site occupancy that would produce the same degree of consistency illustrated here). DC would equal 1 and 0 for Table 2 A and B, respectively. Table 3 summarizes the notation we will be using throughout description of the technique. Table 4 provides a summary of the calculations for distributional consistency using the example data from Table 1, and is intended as a guide for the reader through the remainder of this section.

**Table 2:** Hypothetical survey matrices illustrating a completely consistent, and completely inconsistent distribution of 4 individuals among 16 sites over 4 years.

Time	Site																Popn
	1	2	3	4	5	6	7	8	9	10	11	12	13	14	15	16	Size
A. 'Completely Consistent' Distribution. *DC* = 1																	
**1**	1	1	1	1	0	0	0	0	0	0	0	0	0	0	0	0	**4**
**2**	1	1	1	1	0	0	0	0	0	0	0	0	0	0	0	0	**4**
**3**	1	1	1	1	0	0	0	0	0	0	0	0	0	0	0	0	**4**
**4**	1	1	1	1	0	0	0	0	0	0	0	0	0	0	0	0	**4**
B. 'Completely Inconsistent' Distribution. *DC = 0*																	
**1**	1	0	0	0	1	0	0	0	1	0	0	0	1	0	0	0	**4**
**2**	0	1	0	0	0	1	0	0	0	1	0	0	0	1	0	0	**4**
**3**	0	0	1	0	0	0	1	0	0	0	1	0	0	0	1	0	**4**
**4**	0	0	0	1	0	0	0	1	0	0	0	1	0	0	0	1	**4**

Distributional consistency DC is therefore be 1 and 0 for each of these populations, respectively.

**Table 3:** Definition of terms presented in calculating the index of distributional consistency.

Symbol	Definition
*Y*	Number of time steps (e.g. years) a region was surveyed.
***K***	Levels or degree of consistency with which a site could be used (***k*** * = 2…y*)
*_y_C* ***_k_***	Number of possible combinations of *y* years in which a site could be used to a given level of consistency, ***k***
***List_i,k_***	The list of *_y_C* ***_k_*** possible year combinations, where ***i*** identifies each unique year combination pattern, and so ***i*** = 1…_y_C**_k_** for each level of consistency, ***k***
*M* ***_k_***	Maximum possible instances of consistent site use events for each level of consistency ***k***, given the observed population sizes at each time step.
*S* ***_i,k_***	Smallest population size observed among the years being investigated in a given year combination pattern ***i***.
*O* ***_k_***	Observed instances of sites being used at a given level of consistency, ***k***
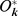	Adjusted value of *O* ***_k_***, to account for *O* ***_k_*** included at higher levels of consistency, **k**
***Q***	List of numbers [***q*** * = *0…(*y-* ***k***)] added to ***k*** to identify higher levels of ***k***
*_(_*_***k****+****q****)*_ *P* ***_k_***	Number of level ***k*** comparisons included in the level (***k+ q***)
*R* ***_k_***	For each level of consistency (***k***), the ratio of observed to maximum possible instances of consistency for the population being considered

‘Years’ is used as a generic time step throughout the text for ease of discussion.

**Table 4:** Example calculation of distributional consistency (*DC*) using the parameters from the example data in Table 1.

*k*	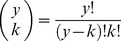	*_y_C_k_*	*List_i_*	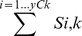	*M_k_*	*_Ok_*	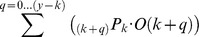	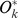	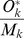	*R_k_*
4	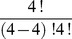	1	1-2-3-4	8	8	1	1(1)	1	1/8	0.125
3	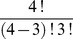	4	1-2-3; 1-2-4; 1-3-4; 2-3-4	8+8+8+8	32	4	1(4)+4(1)	8	8/32	0.25
2	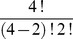	6	1–2; 1–3; 1–4; 2–3; 2–4; 3–4	9+8+8+8+8+8	49	7	1(7)+3(4)+6(1)	25	25/49	0.51
								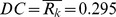		

Grey columns correspond to equations presented in the text, while columns to their left correspond with the associated calculations.

As a standard for comparison, we first calculate the maximum possible degree of consistency to which our observed population *could* have used the array of sites they occupy. We define *y* as the number of time steps (e.g., years or any other relevant interval) that an array of sites was surveyed (e.g., 4 years; Table 1). As discussed above, we define ***k*** as the different possible levels of consistency with which a site could be used (therefore, ***k*** = 2…*y*). For *y* years of surveys, there are a number of ways in which a site could be consistently occupied for each level of *k* (e.g., for ***k*** = 2, a site could be used for two years by being occupied in years one and two, or in years three and four, or any other combination). *_y_C* ***_k_***, the possible ways of comparing *y* years for each level of consistency *k*, is calculated using the standard statistical formula for combinations [11]:
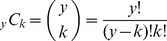
(1)We define ***List_i,k_*** as the list of these *_y_C* ***_k_*** possibilities, where ***i*** identifies each unique year combination pattern (and therefore ***i*** *= *1…*_y_C* ***_k_***; for example, as seen in Table 4, for *y* = 4 and *k* = 3, there are four possible three-year combination patterns (*_y_C* ***_k_*** = 4) and ***List*** *_i,k_* (for *i* = 1 to 4) of these three-year combinations is: 1-2-3; 1-2-4; 1-3-4 and 2-3-4). The survey data (Table 1) indicate how big the population was in each of these years. Next, we calculate the maximum possible instances of consistent site use events (*M* ***_k_***) for each level of consistency (***k***) as:
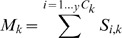
(2)


where *S* ***_i,k_*** is the smallest population size observed among the years being investigated in a given year combination pattern, i (e.g. for ***k*** = 3, if comparing years 2, 3 and 4, the smallest population size *S* ***_i,k_*** is 8 [taken from the data in Table 1, see also Table 4]). We have now calculated the maximum possible consistency the distribution of our population could exhibit, given observed changes in population size, *M* ***_k_***. The next step is to calculate the observed consistency and compare it to this theoretical maximum.

The observed instances of site consistency (*O* ***_k_***) at each level ***k*** are taken from the survey data. The observed instances of consistency from the example data are presented at the bottom of Table 1 (e.g., when ***k*** = 3, *O* ***_k_*** is the number of instances in which sites were consistently used for three years, i.e., *O_3_* = 4 in the Table 1 example). Of course, if a site was used for three years, it was also used for 2 years. This is obvious, however, given the above calculation of maximum possible consistency, *M* ***_k_***, we need to account for the fact that lower levels of ***k*** are intrinsically included in higher levels of ***k***. For example, the level ***k*** = 3 combination of years 1-2-3 intrinsically includes three combinations at the level ***k*** = 2, years 1–2, 2–3 and 1–3. Therefore, to facilitate comparison with our theoretical calculation of maximum possible consistency *M* ***_k_***, the observed level of consistency, *O* ***_k_*** must first be adjusted for consistent site patterns included within higher levels of ***k***. This adjusted *O* ***_k_*** (labelled 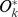) can be calculated as:
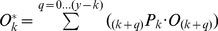
(3)where ***q*** is added to ***k*** to describe all possible higher levels of ***k*** (therefore ***q*** *= *0…[*y-* ***k***]). *_(k+q)_P_k_* is the number of ***k*** level comparisons included in the level (***k*** + ***q***) and, as before, is simply calculated using the standard statistical formula for combinations:



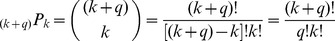
(4)These calculations are also illustrated in Table 4 using the example data from Table 1. For each level of consistency, *k*, we now have a measure of the observed instances of consistency,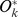 and the maximum possible instances of consistency, *M_k_* given the changes in population size observed among the survey periods. For each level of consistency, ***k***, we can now calculate the ratio of observed to expected consistency***:***

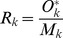
(5)We can now take the final step and calculate our metric of distributional consistency by averaging across levels of consistency, ***k***:
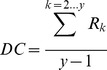
(6)DC is therefore a proportion that represents the degree of consistency in the spatio-temporal distribution of the population across an array of sites at each time step. A value of 0 represents a completely inconsistent spatial distribution, while a value of 1 represents maximum possible distributional consistency among time steps, given the observed changes in population size among years. Like other population metrics (e.g., density, variability, etc.), we are not considering the actual chronology of site occupancy (i.e., a site used in years 1 and 3 will be treated the same as a site used in years 2 and 4). We now have a method to evaluate how consistent distribution patterns are in a given region, which we can compare among different regions or taxa using standard statistical techniques. A Matlab routine for calculating distributional consistency (DC) from any site by time-step survey matrix accompanies this paper as an electronic supplement (Text S1).

### Sites Occupied by Groups of Individuals

For simplicity in the example calculation of DC, a site was considered a territory where individuals were either present or absent. DC is much more general and can be used for a wide variety of ecological scenarios where populations are aggregated in a variety of forms at a range of spatial and temporal scales. For example, in many populations individuals have over-lapping home/foraging ranges, and territorial species frequently aggregate during the non-breeding season. A site could be defined to include overlapping home ranges, large regions of landscape, or any location occupied by groups. For territorial species, it may also be of interest to increase the spatial scale of a site to include multiple territories, depending on the nature of the question of the investigator.

For sites occupied by groups, surveys would indicate the number of individuals present on each site at each time step (e.g., Table 5). As before, the number of possible instances of consistent occupancy (M*_k_*) is determined by the number of individuals in the population. To calculate the observed instances of consistency *O_k_* we now need to consider that a site can be occupied by more than one individual. To do this, we calculate how many times the same numbers of individuals were observed at each site. For example, if 2 individuals were observed at a site in years 1 and 2, and 3 individuals were observed in year 3, there would be 2 observed instances of 3-year consistency (i.e., O*_k_* = 2 for *k* = 3). Table 5 provides several hypothetical examples for a study system where sites can be occupied by groups. For instance, at site 5, although there was 1 sighting in year 1 and 2 sightings in year 2, there is only 1 instance of 2-year consistency (*O_k_* = 1 for *k* = 2). At site 12, there were 3 sightings in year 1, 2 in year 2 and 1 in year 3: therefore there is 1 instance of 2-year consistency and 1 instance of 3-year consistency. Instances of 2-year consistency included within the 3-year consistency are accounted for by the metric (see above). It can now be understood that the previous territorial example was simply a special case of how individuals occupy sites, and the calculation of DC proceeds in exactly the same way, treating each individual sighting as a case for which consistency is evaluated. In this manner, DC can be used generically for any spatiotemporal definition of ‘sites’ or ‘region’, allowing its application and comparison among populations exhibiting any form of structure or dynamics.

**Table 5:** Hypothetical survey matrix of the number of individuals at each site, in each time step for a situation where groups of individuals can occupy a site.

Time	Site																Popn
	1	2	3	4	5	6	7	8	9	10	11	12	13	14	15	16	Size
**1**	1	0	0	0	1	2	1	2	0	0	0	3	1	2	0	3	**16**
**2**	0	2	0	0	2	1	0	0	2	3	0	2	3	0	2	3	**20**
**3**	0	0	3	0	0	0	3	0	1	0	2	1	0	3	2	3	**17**
**4**	0	0	0	1	0	0	0	2	0	1	3	0	2	2	2	3	**15**
**SUM**	1	2	3	1	3	3	4	4	3	4	5	6	6	7	6	12	
**Summary of Observed Consistency**																	**O_k_**
***k*** ** = 2**	*0*	*0*	*0*	*0*	*1*	*1*	*1*	*2*	*1*	*1*	*2*	*1*	*1*	*0*	*0*	*0*	***11***
***k*** ** = 3**	*0*	*0*	*0*	*0*	*0*	*0*	*0*	*0*	*0*	*0*	*0*	*1*	*1*	*2*	*2*	*0*	***6***
***k*** ** = 4**	*0*	*0*	*0*	*0*	*0*	*0*	*0*	*0*	*0*	*0*	*0*	*0*	*0*	*0*	*0*	*3*	***3***

As per Table 1, population size in each time step is the sum of each row. Below the main table, the column sums and a summary of the number of instances consistency was observed at each level, k are presented for each site to illustrate how distributional consistency *DC* is calculated.

## Results

### Multi-scale Analysis

The distributional consistency DC technique can be calculated for any definition of sites and regions desired. A site is simply the finest spatial resolution (grain) the researcher chooses to consider and could be defined as a point count station, a nest-site, discrete habitat block, a defined stretch of shoreline, etc. The region, in turn, is an assemblage or array of sites over which the DC index is calculated (extent). A given study area might include several regions among which DC could be compared. DC can therefore be used in a wide variety of contexts, which could include comparing distribution patterns of different species, or evaluating how distribution patterns change across spatial and temporal scales.

Defining a patch is an issue in its own right and requires careful consideration of multiple scales [13]. In many cases, habitat patches are not discrete entities. The definition of both a site and region will depend on the nature of the question being asked, and should always be selected with careful consideration of a species’ natural history characteristics. For example, as discussed in the examples above, the definition of a site could depend on the species’ degree of territoriality or aggregation patterns at particular stages of the annual cycle. Further, the definition of a site or region need not be kept constant for a given study, and it could be valuable to consider how DC varies across a range of spatial scales. A hierarchy of spatial scales are important in determining both the structure of patches and the resulting habitat selection decisions of individuals [13]. Research results and the relevance of ecologically important factors often depend on the scale of analysis [14]–[18]. It could therefore be informative to calculate DC over a range of spatial scales by changing the scale at which sites and/or regions are defined. A species’ natural history characteristics and distribution will determine the finest (grain) and largest (extent) scales of relevance, which could change throughout stages of the annual and life cycles.

**Figure 1:**
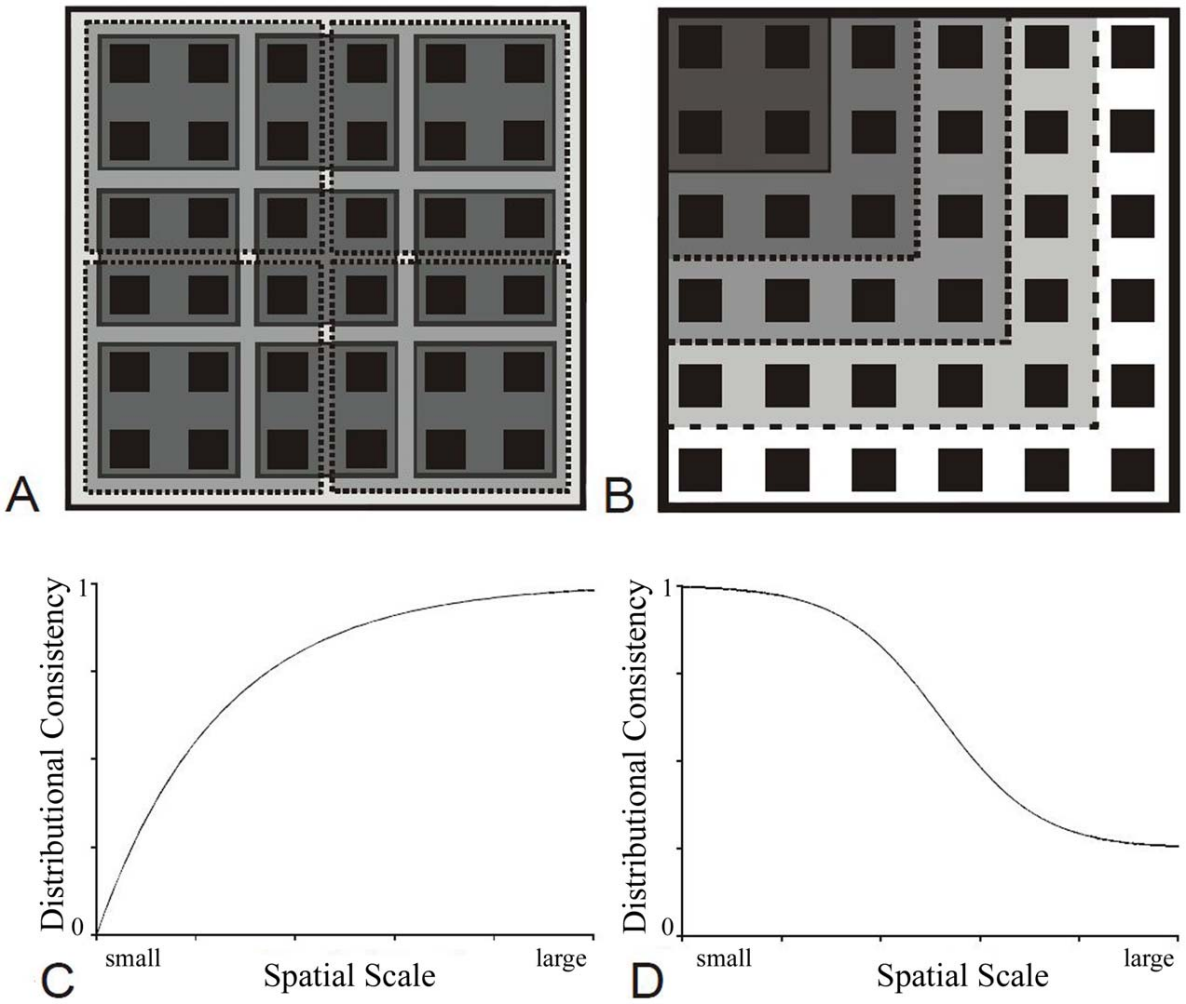
Schematic representation of the effects of changing the spatial scale on Distributional Consistency. Changing the scale of (A) a site or (B) the region can influence observed distributional consistency patterns (C and D, respectively). Increases in spatial scale follow a gradient from darker to lighter squares. As the spatial scale of a site (grain) approaches that of a region (extent - C), distributional consistency will approach a value of 1. When the scale or extent of the region is extended (D) to include more sites, distributional consistency could reach an asymptotic value describing consistency of the distribution across the species’ range. In both scenarios, the shape of the spatial scale versus distributional consistency curve will be the feature of interest for evaluating the scales of movement relevant to the species and question being considered.

Figure 1a schematically illustrates holding the region (largest square) constant, and changing the scale of a site. DC should generally increase as the spatial scale of a site increases (Fig. 1c), approaching a value of 1 as the scale of a site approaches that of the region. In contrast, keeping the scale of a site constant and changing the scale of the region (Fig. 1b) essentially equates to considering a larger or smaller geographic study area and therefore including a different number of sites. Under this scenario, spatial consistency would again equal 1 when sites and regions are equivalent in size, and should be expected to decrease as more sites are included (e.g., Fig. 1d). In each case, the specific shape of the distributional consistency versus spatial scale curve could be an interesting feature of investigation. For example, sharp changes in this relationship could be useful in defining the domains of scale across which movement decisions are important for a given species. In this manner, it may even be useful to define regions or other demographic units based on an analysis of DC across multiple spatial scales. Similarly, the time scale could be changed, and/or DC could be compared among different temporal periods. DC can therefore provide an important and flexible multi-scale tool for analysing spatiotemporal distribution patterns. Kotliar and Wiens ([13] and references therein) provide a good starting point for further reading on defining patches across multiple scales of investigation.

### Robustness to Survey Error

Survey error is an important consideration for metrics of population characteristics and proper inferences cannot be made without considering issues of detectability [12]. DC considers only observed population size at each time step, therefore any individuals missed in the survey are not assessed for their consistent occupancy patterns, making DC conservative to survey error, particularly if the error rate is consistent across sites within each region. Of course, if there is high heterogeneity in the habitat characteristics and survey error of individual sites within a given region, then dealing with survey error will be problematic and must be dealt with on a case by case basis. These kinds of extreme detection issues wreak havoc with all metrics derived from survey data (habitat use, trends, etc), so this is not a problem unique to DC and it is up to the users of survey data to ensure the data were collected in a reasonable manner for the desired use, something that survey biologists routinely address. Under less extenuating circumstances, if survey error is relatively consistent within a region, then on average a consistent proportion of individuals (but not necessarily the same ones) will be missed at each site, and so the measure of distributional consistency will be relatively unaffected. As we will illustrate, this allows robust comparisons of DC, even among species or regions that differ fairly substantially in survey error rates.

**Figure 2:**
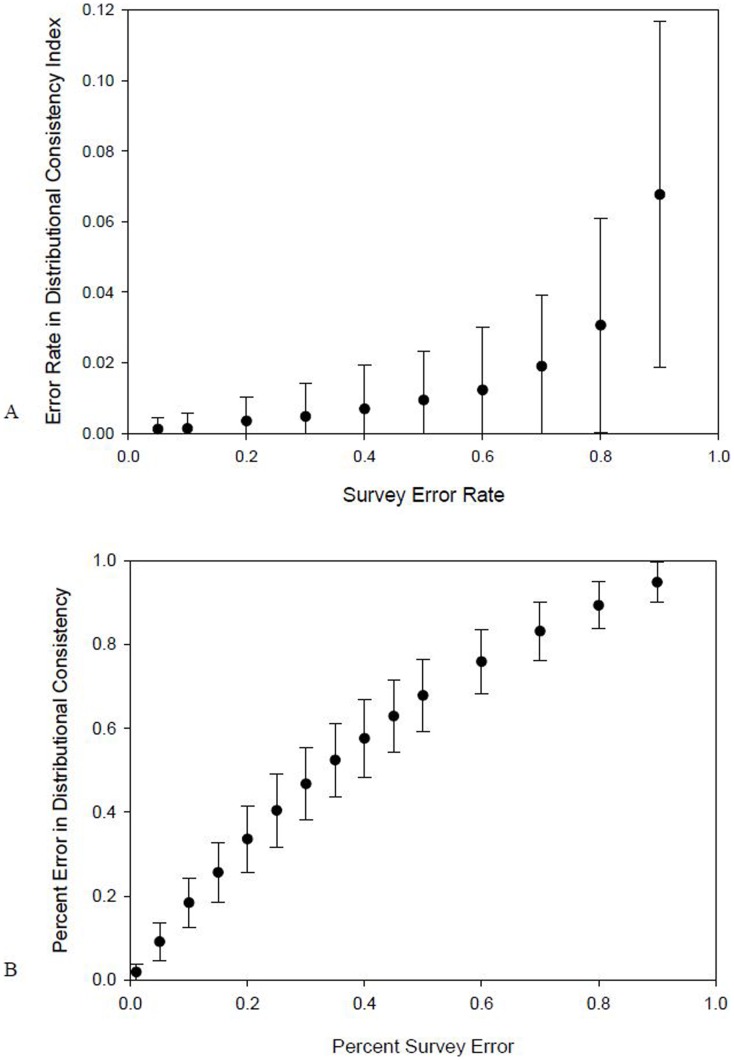
The influence of survey error on measures of distributional consistency. For situations where groups of individuals can occupy sites (A), simulations of survey error indicate that distributional consistency DC scores will be influenced by less than 5%, even up to survey error rates of 80%. For territorial species presence/absence data (B), the degree of survey error affects measurement of DC, however this influence is highly predictable and can therefore be easily corrected for. Error bars are standard deviations.

To quantitatively demonstrate the robustness of DC to survey error, we simulated fifty 5-year distribution patterns within a region containing 50 sites, with a random number of individuals (range 0 to 250) at each site, in each year. This provided a wide range of distribution patterns of different population sizes and consistency (DC). For each distribution, survey error was simulated 5 times by selecting random individuals to be missed in each year at error rates from 5 to 90%. For each distribution pattern, DC was evaluated for the true population, and at each rate of survey error. Even at 80% survey error, DC was affected by less than 5% (figure 2a). This is because only observed individuals are considered in calculating DC. This is a particularly appealing attribute and indicates DC will be robust to a wide range of survey error, even if survey error differs drastically among regions and species.

The case for territorial species is a special one, as observations for each site are binary presence/absence instead of continuous numbers of individuals (note that if only a single pair is available for detection at a site, this could have a considerably different effect on survey error than the case when presence/absence data is used when multiple individuals occupy a site). While 10% survey error within a region also means a 10% probability of missing individuals at each site, instead of reducing population size by 10% on average at each site, a territory holder will be either missed or not in each year, which could have an influence on DC measurements. To evaluate this effect, we simulated one hundred 5-year distribution patterns for a territorial species presence/absence data, with a random overall population size in each year (range 25–75 individuals), in a region with 100 territories (sites). For each distribution, we randomized survey error 15 times for each error rate (range 1–90% survey error) and calculated DC for the true population and each randomization. As indicated in Figure 2b, DC changed substantially with survey error for territorial species presence/absence data, however this relationship was highly predictable (R^2^ = 0.996, df = 4,745, p = 0.00). Therefore, when territorial presence/absence data are used, DC can be easily corrected for differences in survey error among regions or taxa, and robust comparisons can still be made. The regression equation describing this predictable relationship can be derived for a given study by conducting similar simulations across different survey error rates, and using the same number of territories and survey years being investigated. Of course, caution should be employed, particularly for presence/absence data when survey error is unknown.

## Discussion

### Applications for Distributional Consistency - *DC*

Typically, measures of consistent site use have been drawn from repeated observations of marked individuals. Considerable insights will continue to be obtained from capture-mark-recapture (CMR) approaches, though there are a number of ways in which DC could enhance CMR analyses. Research logistics often dictate that marking and resighting efforts are focussed on small areas with high animal densities, which may not be representative of the population (particularly given e.g. source-sink dynamics; [19]). Additionally, mark-recapture and telemetry methods require situations in which one can handle animals, mark them in a manner that does not influence their behaviour or mortality, and have a high likelihood of detection [20]. While these limitations are recognized, DC provides a technique to measure fidelity patterns at higher levels of organization than the individual, which could be very informative and complimentary when used in conjunction with existing CMR techniques. In the following section we describe how DC could be used to make inferences about the movement and habitat use decisions of individuals within a region.

### Linking Individual Movements and Distribution Patterns

**Figure 3:**
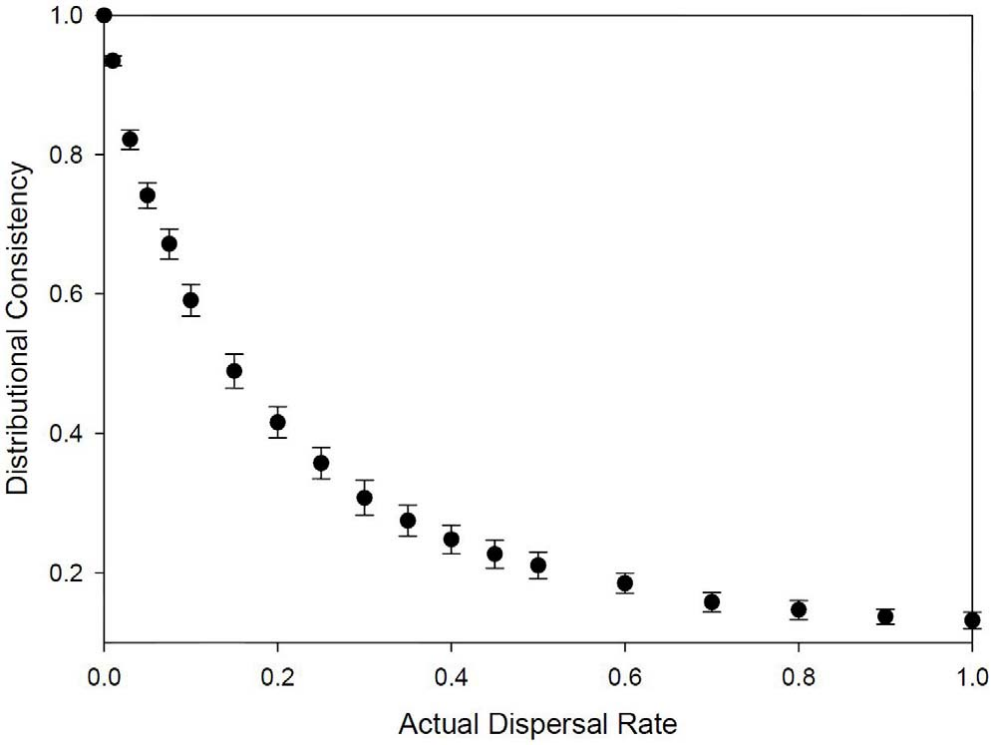
Results of tracking individual dispersal in simulations as described in the text, illustrating a strong relationship between individual dispersal decisions and consistency in the regional distribution *DC* (error bars are standard deviations). This indicates *DC* provides a useful estimate of dispersal rates from basic survey data of unmarked individuals.

Understanding fidelity decisions of *individuals* requires studies of marked individuals. However, because individual decisions cumulatively influence distribution patterns, DC is a fundamental consequence of fidelity and habitat choice. High fidelity will produce very consistent site use patterns and DC. Additionally, low DC implies low fidelity. In these situations, DC can be used to make direct inference to individual movement patterns, from simple analysis of survey data. We can illustrate the functional relationship between individual dispersal decisions and population level distributional changes by conducting simulations of known dispersal rates, and measuring the resulting DC. We simulated a population of 100 individuals that were initially randomly distributed within a region containing 100 sites. For each rate of dispersal (range 0–100%), random individuals in the population were selected and dispersed to new sites (randomly selected) within the region, at each time step for 25 years. 50 simulations were conducted for each dispersal rate, and DC was calculated over the last 10 years of each simulation. As indicated in Figure 3, DC reflects the dispersal rate of individuals to a high degree of precision (regression equation: 1/DC = 1.061+7.119*(dispersal rate); R^2^ = 0.976, df = 948, p<0.0001). Although the relationship is non-linear (due to site swapping, discussed below), the extremely high R^2^ indicates that it is highly predictable.

Although high fidelity will produce very consistent site use patterns and DC, and low DC implies low fidelity, the inverse of these two statements, i.e., that low fidelity leads to low DC, and that high DC implies high fidelity, both depend on the degree of site swapping among individuals, and therefore require more careful consideration. Although site swapping could occur in a purely random fashion, individual movement decisions are likely to depend on the decisions of others, the costs and benefits of various options, available information, age and prior experience. Territorial individuals, for example, can follow an ideal despotic distribution [5], whereby the death or emigration out of the region by a high quality individual vacates a site, and all individuals shift territories in accordance with their social status. The influence of change in social structure on site swapping will likely depend on the level at which change in the social hierarchy takes place (i.e., if sites are occupied based on rank, mortality of a low ranking individual will lead to less site switching than mortality of an alpha individual). In non-territorial species, multiple individuals can occupy a site among years, however, density and frequency dependent processes presumably influence movement and fidelity at the site level in a similar manner to territorial species (i.e., whereby death or emigration decreases population size below carrying capacity of the site, allowing new individuals to settle). In a closed system, for a fixed number of sites and random dispersal, the more individuals that are present, the more site swapping will occur by chance. Therefore, while the shape of the DC and dispersal rate relationship will be consistent with Figure 3, because of increased site swapping, DC will vary over a narrower range per dispersal rate for populations of larger size. As an extreme example, if we allow density dependence at the site level, limiting the maximum number of individuals per site, and set the population size equal to the carrying capacity, then the only option for dispersal is site swapping, and the distribution will be consistent across years, despite differences in dispersal rate. Therefore, if there is a high degree of site swapping, i.e. low fidelity to specific sites by individuals, but the overall population uses the same array of sites, then although high DC will not always imply high fidelity, it will still appropriately reflect the consistent use of a presumably important set of sites (i.e., habitat) by the population. Random site swapping is likely a rare occurrence in nature. This is an area that requires further empirical investigation, because in many studies investigating return rates of marked individuals, it is unknown what happens to individuals following a dispersal event, and because the marked individual is the unit of study, it is rarely determined how new individuals fill the vacated site.

Although the specifics of the study system, particularly the degree of density dependence and site swapping should be carefully considered, the highly predictable relationship between DC and dispersal rate is a very appealing result. Estimation of dispersal rates using marking and re-sighting techniques often requires a high degree of logistical effort, that in some situations is not feasible. In such situations, DC could provide an important tool to index dispersal rates of individuals, using population level distribution patterns from standard surveys of unmarked individuals. In a recent example analysis [21] DC was quantified in conjunction with several other approaches including individually marked birds with radio telemetry techniques and survey data. This analysis allowed evaluating the effectiveness of DC by comparing it with data from marked birds.Results revealed a strong relationship between DC, individual movements and foraging site fidelity across different habitats and time scales, indicating that DC can provide important inferences that will be useful in studying the relationship between individual movements and distribution patterns. While radio-telemetry and capture-mark-recapture studies require extensive time and financial costs to implement, this example analysis indicates that DC can provide reliable estimates of movements and fidelity from simple survey data.

For many species, both surveys and mark-recapture studies are frequently conducted as a part of basic monitoring and management, though interrelationships between these techniques are rarely considered. These results indicate it will be particularly exciting to investigate how survey data analyzed using DC correspond to mark-recapture results within and between surveyed regions and many existing data sets are amenable to such investigations. Existing GIS data sets also could allow investigation of how individual movements and DC are related to habitat quality and heterogeneity, inter-site and inter-region distances, and the clumping/dispersion of sites.

Landscape ecology research has indicated that both within region (between site) movement and dispersal between regions can change with a regional population’s reproductive output, e.g. [15], [19], while individual marking research has also shown fidelity/dispersal decisions can be related to individual reproductive success, e.g. [6], [22], [23]. This is encouraging as processes occurring at the level of individuals should be expected to percolate across levels of organization to overall population dynamics and provide the mechanisms for population change, e.g. [24]. The complexity of ecological processes is an important area requiring a great deal of research and it is intended that distributional consistency can contribute to this effort in the context of movement decisions and habitat choices.

### Habitat Use and Quality

The decisions individuals make about reusing or moving to new sites are expected to be related to the relative costs and benefits in each habitat, e.g. [5]. In northern Labrador, DC was strongly related to local population structure, density and stability of Harlequin Duck populations, and negatively correlated with the regional distribution of avian predators [25], [26]. The link to habitat quality is an important aspect of consistency in distributions, as sites with lower cost:benefit ratios should be used more consistently. Typically, survey data have been linked to habitat quality in terms of biophysical habitat features or variation in density or abundance, which alone may not accurately reflect habitat quality [27]. Sergio and Newton [10] provided evidence that frequency of occupancy (which can also be interpreted as the degree of consistency in use of a site by the *population*) is strongly related to numerous components of territory quality. Additionally, consistent site use by *individuals* (fidelity) has been related to reproductive success [6], [22], [23]. Considering these arguments at a larger spatial scale, regions in which sites are used consistently may well be of higher quality than similar regions containing sites which are used less consistently. Measuring distributional consistency could therefore provide information about the value of habitat at a regional level, allowing more informed conservation management decisions.

Processes occurring at spatial scales larger than the region can be particularly important for understanding habitat use and demographic structure. For example, if the population has a source-sink structure [19], different habitat and movement decisions of individuals could produce considerable differences in the consistency of distribution patterns among regions. In this context, comparison of DC among regions could provide insight into differences in demographic characteristics and the regional value of habitat patches. Such comparisons will require consideration of other demographic parameters. For example, although DC is mathematically independent of regional population size, it is likely it will be ecologically related in so far as higher density populations reflect populations closer to carrying capacity. That is, if population size is consistently close to the number of suitable sites in a region, then DC will be high as all sites are always occupied. Therefore, while DC reflects how the habitat is used, it does not reflect how much habitat is available, and both large and small regions could be relatively important, and used to the same degree of consistency. DC therefore provides an informative tool for comparisons among regions that can facilitate the interpretation of other demographic indices, such as population density and variability/stability [e.g. 28], which can be calculated from the same basic survey data.

### Summary and Implications

DC promises to be an important tool for quantifying consistency in the distribution patterns of populations. Furthermore, it provides a framework to investigate the relationship between decisions of individuals and larger scale population and landscape processes, and for relating spatio-temporal distribution patterns to various physical and biological attributes of ecosystems. A recent example analysis (21) indicates DC corresponds well with more intensive and costly radio telemetry mark-recapture approaches. It is particularly appealing in that it can be calculated from simple survey data of unmarked individuals, which will make it easy to incorporate with analysis of other spatial and temporal components of a species’ distribution, such as population size (or density) and variability. In conjunction with these metrics, DC has the potential to be a useful tool for conservation and management by providing further rigour to identification of key habitats and areas of demographic significance.

## Supporting Information

Text S1 A Matlab.m file provided online allows calculating ‘Distributional Consistency’ from an input site (columns) by time interval (rows) matrix of survey data.
(M) Click here for additional data file.
